# Identification of novel drug-specific PARP inhibitor resistance mechanisms in ovarian cancer–implications for clinical practice

**DOI:** 10.1038/s41416-026-03423-z

**Published:** 2026-04-17

**Authors:** Caitlin J. Macdonald, Amber McWhirter, Aparajitha Vaidyanathan, Michelle J. Ferguson, H. Christian Eberl, Euan A. Stronach, Lynne Sawers, Gillian Smith

**Affiliations:** 1 Division of Cancer Research, School of Medicine, University of Dundee, Dundee, UK; 2 Tayside Cancer Centre, NHS Tayside, Dundee, UK; 3 Cellzome, A GSK Company, Heidelberg, Germany; 4 Research Technologies, GSK, Stevenage, UK; 5 Present Address: Division of Paediatric Oncology, Johns Hopkins University School of Medicine, Baltimore, MD USA

**Keywords:** Ovarian cancer, Molecular medicine

## Abstract

**Background:**

Maintenance PARP inhibitor (olaparib or niraparib) treatment is commonly prescribed following carboplatin/paclitaxel chemotherapy in ovarian cancer patients, but response is compromised by adaptive drug resistance [1]. We have shown that P-gp/*ABCB1* influences resistance to paclitaxel and olaparib, but similar niraparib resistance mechanisms have not been described [2, 3].

**Methods:**

We used qRT-PCR, Western blot, RNASeq and LC-MS/MS proteomics analysis to compare drug transporter expression in sensitive and resistant immortalised and primary patient-derived cell lines. *ABCB1* and *ABCG2* expression was modified by shRNA-mediated knockdown and heterologous expression, with chemosensitivity changes assessed by MTT and clonogenic assays. Substrate specificity of P-gp and BCRP was assessed by efflux assays in polarised cells.

**Results:**

P-gp/*ABCB1* expression was not increased in A2780nirapR cells, which alternatively up-regulated BCRP/*ABCG2*. *ABCG2* was consistently induced in niraparib-resistant patients, but *ABCB1* only in patients pre-treated with paclitaxel. sh*ABCG2* re-sensitised A2780nirapR cells, while heterologous expression in A2780 cells induced drug resistance. Efflux assays confirmed that olaparib and niraparib are both P-gp and BCRP substrates, suggesting that resistance results from transcriptional regulation of efflux transporters not substrate specificity.

**Conclusions:**

Treatment-induced BCRP/*ABCG2* induction is a novel clinically relevant niraparib resistance biomarker. Routine inclusion of paclitaxel in first-line chemotherapy regimens may promote efflux transporter-mediated resistance, compromising response to PARPi maintenance treatment.

## Background

High-grade serous ovarian cancer, the most common and aggressive histotype, is frequently diagnosed when already advanced (FIGO Stage III/IV, https://www.cancerresearchuk.org/health-professional/cancer-statistics/statistics-by-cancer-type/ovarian-cancer#ovarian_stats4). Surgery is not always possible in patients with advanced disease, and response to neo-adjuvant or adjuvant chemotherapy, most commonly combining carboplatin and paclitaxel, influences both progression-free and overall survival [4]. First-line chemotherapy is often extended by maintenance PARP inhibitor (PARPi, olaparib or niraparib) treatment, with eligible patients selected following assessment of germline and/or somatic *BRCA1/2* mutation status or by extended homologous recombination deficiency (HRD) panel testing [5, 6]. HRD status is an important biomarker of PARPi response, as PARPi-mediated inhibition of poly (ADP-ribose) polymerase 1 (PARP1) limits the ability of cells to repair single strand DNA breaks [7] and leads to PARP trapping and replication fork stalling [8]. Resulting double strand breaks are preferentially repaired by homologous recombination-mediated repair mechanisms, but this process is compromised in *BRCA1/2* mutant or HRD deficient cancers, which exhibit synthetic lethality with DNA damaging platinum drugs and PARPis [9].

Although many patients initially respond well to treatment, the development of chemotherapy-induced resistance is a common, treatment-limiting complication [10]. Various resistance mechanisms including reduced drug bioavailability resulting from down-regulation of the copper import transporter 1 (CTR1, [11] and up-regulation of the detoxifying glutathione S-transferase GSTP1 [12] limit carboplatin bioavailability, while resistance to paclitaxel results from altered binding target β-tubulin isoform expression [13] or, more commonly, chemotherapy-induced increased expression of P-glycoprotein, a membrane-bound ATP-dependent efflux pump, encoded by *ABCB1* (MDR1) [14]. The development of multidrug resistance is a particular clinical challenge as it not only limits response to initially prescribed drugs, but to structurally diverse alternatives which are also P-gp substrates [15]. We have previously shown that A2780 ovarian cancer cells made resistant to paclitaxel are cross-resistant to olaparib, with increased P-gp expression promoting resistance to both drugs [16]. Importantly, in contrast to previously reported mechanisms of PARPi resistance including acquisition of reversion mutations to restore HR pathway function [17], our data highlights that the impact of increased P-gp expression is PARPi-specific, with olaparib and rucaparib cross-resistant in A2780pacR cells, while sensitivity to veliparib and AZD2461 is not influenced by P-gp expression.

An alternative PARPi, niraparib (Zejula™), was licensed for first-line maintenance treatment of ovarian cancer patients, following the NOVA [18] and PRIMA [19] clinical trials. Niraparib is licensed for all platinum-sensitive patients, independent of *BRCA1/2* mutation status although, like olaparib, exhibits greater efficacy in HRD patients. Our previous data highlights the importance of assessing whether niraparib, like olaparib, is a P-gp substrate and therefore also influenced by paclitaxel-induced increased drug efflux. Importantly, identification of additional novel, niraparib-specific resistance mechanisms may facilitate extension of current biomarker-guided prescribing protocols to limit the development of drug resistance in ovarian cancer patients.

## Methods

### Ethical approval

The Dundee Ovarian Cancer Study (DOCS) was approved by the East of Scotland Research Ethics Service REC1 Committee (13/ES/0035). Informed consent was obtained from all participants, and the study performed in accordance with the Declaration of Helsinki.

### Cell lines and cell culture

A2780 cells were obtained from the European Collection of Authenticated Cell Cultures (ECACC). Novel niraparib-resistant (A2780nirapR) and olaparib-resistant (A2780olapR, [16]) A2780 derivatives were created following continuous incremental drug selection (0.1 µM–12.8 µM niraparib; 1 µM–20 µM olaparib), to mimic typical patient peak plasma concentrations [20]. Cell lines were authenticated by short tandem repeat profiling (Centre for Life, Newcastle, UK) and routinely tested for mycoplasma contamination (Lonza Biologics, Slough, UK).

Cell lines were cultured in RPMI-1640 medium supplemented with 10% v/v foetal bovine serum (Thermo Fisher, Renfrewshire, UK) and 1% v/v penicillin/streptomycin (Sigma Aldrich, Dorset, UK). Media for resistant cell lines was supplemented with 12.8 µM niraparib or 20 µM olaparib every third passage to maintain resistance.

Ascites samples were collected from consented DOCS study patients following paracentesis, transferred to a Category II cell culture facility and mixed 1:1 with ascites media (1:1 MCDB (Sigma Aldrich, Dorset, UK)/Media 199 (Gibco, Renfrewshire, UK), 10% v/v FBS, 1% v/v penicillin/streptomycin. Cells were incubated for 3–5 days before differential trypsinisation to limit fibroblast contamination, then primary cells incubated for an additional 3 days before use in subsequent experiments.

### MTT chemosensitivity assays

MTT (3-(4,5-dimethylthiazol-2-yl)-2,5-diphenyltetrazolium bromide) chemosensitivity assays [21] were used (3 independent experimental repeats) to compare the sensitivity of A2780, A2780nirapR and A2780olapR cells to niraparib, olaparib, carboplatin, paclitaxel, camptothecin, irinotecan and the *ABCG2* inhibitor Ko143. Cells were seeded at 5000 cells/well and treated in triplicate with 2-fold serial dilutions of each drug (all sourced from Selleckchem, Hertfordshire, UK) - 1.25 µM–40 µM niraparib, 1.56 µM–50 µM olaparib, 2.66 µM–85.12 µM carboplatin, 1 µM–31.85 µM paclitaxel, 0.16 µM–5 µM camptothecin and 0.31 µM–10 µM irinotecan. Ko143 concentrations (0.06 µM–2 µM) were initially tested in A2780nirapR cells before subsequent experiments combined 1 µM Ko143 with serial dilutions of either niraparib or olaparib. Cells were incubated with drug or DMSO vehicle control for either 144 h (niraparib and olaparib) or 72 h (carboplatin, paclitaxel, camptothecin and irinotecan), before removal of media and incubation with 100 µL 0.5 mg/mL MTT solution in phenol red-free RPMI for 3 h at 37 °C. MTT solution was removed and 100 µL DMSO added to solubilise formazan crystals, before absorbance was quantified at 570 nm using a spectrophotometer (MultiSkan, ThermoFisher Scientific). Percentage of viable cells remaining following drug treatment was calculated relative to vehicle-treated control cells and associated EC_50_ values estimated from log dose-response curves using Prism 10 software (GraphPad Software, Inc., La Jolla, CA, USA).

### RNA extraction

Total RNA was extracted from 1 × 10^6^ cells using RNeasy Mini Kits (Qiagen, Manchester, UK), following the Manufacturer’s instructions, including an on-column DNase digestion (RNase free DNase Kit, Qiagen, Manchester, UK). RNA yield and integrity were confirmed using a Nanodrop ND1000 spectrophotometer (Thermo Fisher, Renfrewshire, UK).

### qRT-PCR analysis

RNA (200 ng) was reverse transcribed into cDNA using TaqMan Reverse Transcription Reagents (Thermo Fisher, Renfrewshire, UK) according to the Manufacturer’s instructions, replacing oligo dT with random hexamers. *ABCG2* (Hs01053790_m1), *ABCB1* (Hs00184500_m1) and *18S ribosomal RNA* expression (438839) was assessed in 20 µL reactions, combining 10 µL TaqMan universal master mix (Thermo Fisher, Renfrewshire, UK), 1 µL gene-specific probe, 1 µL cDNA and 8 µL nuclease-free water. Each reaction was performed in triplicate and run on the standard PCR programme (50 °C 2 min, 95 °C 10 min, and 40 cycles of 95 °C 15 s, 60 °C 1 min) on a QuantStudio5 qRT-PCR instrument (Thermo Fisher, Renfrewshire, UK). Baseline and threshold values were calculated automatically and gene expression quantified by cycle threshold (Ct) values, with relative gene expression comparing Ct values in target gene and 18S rRNA control (ΔCt), as previously described [16]. Compound errors (s) were calculated using the formula *s* = ((standard deviation target gene)^2^ + (standard deviation 18S rRNA)^2^)^½^ (https://assets.thermofisher.com/TFS-Assets/LSG/manuals/cms_042380.pdf).

### shRNA-mediated gene knockdown

shRNA constructs packaged in pLKO.1 vector were purchased as bacterial glycerol stocks (SigmaAldrich), and plasmid DNA extracted using a QIAGEN HiSpeed maxi kit, according to the Manufacturer’s instructions. 2.5 × 10^5^ cells were transfected with 2 µg *ABCG2* shRNA construct (TRC0000059802) or empty-vector control (pLKO1^EV^), using 5 µL Lipofectamine 3000 and 4 µL P3000 reagent, in serum-free OptiMEM medium (all sourced from Thermo Fisher, Renfrewshire, UK). After 24 h, cells were re-seeded into 10 mm cell culture dishes, using fresh media containing 8 µg/mL puromycin for 120 h. Surviving colonies were selected using 150 µL colony chambers (Merck, Dorset, UK) and grown in selective puromycin-containing media until confluent, before harvest for mRNA and protein confirmation of gene knockdown.

### RNASeq analysis

RNA was extracted in duplicate from A2780, A2780olapR and A2780nirapR cells and from paired drug-sensitive and drug-resistant DOCS study patients, as previously described and sent to the Genetics Core, Wellcome Trust Clinical Research Facility, Edinburgh (https://clinical-research-facility.ed.ac.uk/core-services/genetics/sequencing) for confirmation of RNA integrity using an Agilent 2100 Bioanalyser, with subsequent library preparation and RNA sequencing analysis performed using Ion Ampliseq Transcriptome Human Gene expression technology (ThermoFisher Scientific). Subsequent bioinformatics analysis was performed by FIOS Genomics (https://www.fiosgenomics.com). Quality of sequencing reads was assessed using FastQC and reads aligned to the human genome using STAR aligner. Sample data was normalised using trimmed mean of M-values normalisation and expression values transformed using voom, with Principal Component Analysis (PCA) plots used to illustrate global gene expression profiles. Significant differentially expressed genes (DEGs; fold change threshold≥2) were identified in pairwise comparisons, with *p*-values corrected for multiple testing using the Benjamini-Hochberg false discovery rate (FDR) adjustment (FDR-adjusted p ≤ 0.05; [22]). DEGs were represented in volcano plots, plotting log_2_ (fold-change) against -log10 (adjusted *p*-value). Functional enrichment analysis was performed on pairwise comparisons of all significantly expressed genes, comparing KEGG pathways and GO terms.

### Over-expression of *ABCG2* in A2780 cells

2.5 × 10^5^ A2780 cells were seeded per well of a 6-well plate and incubated for 24 h, before transfection with 2 µg pCMV3.1-*ABCG2* plasmid or pCMV3.1^EV^ control plasmid (both sourced from SinoBiological, Eschborn, Germany) using 5 µL Lipofectamine 300 and 4 µL P3000 reagent in serum-free OptiMEM medium (all sourced from Thermo Fisher, Renfrewshire, UK). Cells were incubated for 24 h before re-seeding into 10 cm^2^ dishes using fresh media containing 50 µg/mL hygromycin. Single cells were grown into individual colonies and expanded separately until confluent, prior to confirmation of *ABCG2* mRNA and protein expression.

### Protein extraction

Protein was extracted from 1 × 10^6^ cells by scraping in 150 µl RIPA buffer (50 mM Tris-HCl, pH 8.0, 150 mM NaCl, 0.1% SDS, 0.5% sodium deoxycholate, 1% NP-40, 2 mM EDTA) containing protease inhibitor cocktail (SigmaAldrich). Lysates were centrifuged at 2000 × *g* for 5 min to pellet cellular debris and the resulting supernatant used in a Detergent Compatible (DC) assay (BioRad, Hertfordshire, UK) to determine protein concentration relative to a bovine serum album standard curve (0–2 mg/mL).

### Western blotting

Samples were diluted to 1 µg/µL with RIPA buffer, mixed with 10 × sample reducing agent (500 mM dithiothreitol) and 4 × BOLT sample buffer (both Thermo Fisher, Renfrewshire, UK) and denatured at 100 °C for 5 min, prior to separation by gel electrophoresis using 10% SDS-PAGE gels in Tris-glycine buffer (25 mM Tris, 192 mM glycine, 0.1% SDS, pH 8.3) with 0.5 mL antioxidant (Thermo Fisher, Renfrewshire, UK), before transfer onto 0.45 µM nitrocellulose membrane (Thermo Fisher, Renfrewshire, UK) in Tris-glycine-methanol buffer (25 mM Tris, 192 mM glycine, 20% v/v methanol). Non-specific antibody binding was blocked by incubation for 30 min in TBS-T (50 mM Tris-HCl, pH 7.9, 150 mM NaCl, 1% Tween-20) containing 5% milk powder, before overnight incubation with either BCRP rabbit monoclonal antibody (1:1000, Abcam, ab207732), P-gp mouse monoclonal antibody (1:1000, SigmaAldrich, MAB4120) or β-actin mouse monoclonal antibody (1:1000, SantaCruz, sc-47778). Membranes were washed in TBS-T before incubation in either HRP-goat anti-rabbit polyclonal secondary antibody (1:2000, BioRad, #1706515) or HRP-goat anti-mouse polyclonal antibody (1:2000, BioRad, #1706516). Blots were developed using an ECL chemiluminescence kit (Sigma Aldrich, Dorset, UK) using Cytiva Hyperfilm (FisherScientific, Leicestershire, UK).

### Proteomics analysis

1 × 10^6^ cells were seeded per 10 cm^2^ culture dish and incubated for 48 h before protein harvest by cell scraping using 300 µl SDS-lysis buffer (50 mM Tris-HCl pH 7.4, 1.25% SDS, 1 mM MgCl_2_, 2 U/µL benzonase nuclease, EDTA-free protease inhibitor cocktail). Lysates were heated to 95 °C for 5 min, using a thermomixer at 900xRPM (StarLab), cooled to room temperature and 3.44 µL benzonase (≥250 units/µL Sigma Aldrich, Dorset, UK) added, incubated at 37 °C using a thermomixer at 900xRPM for 30 min, centrifuged at 20,000xG for 20 min and supernatants snap frozen in liquid nitrogen and stored at −80 °C. Samples were further processed following a modified version of the single pot solid-phase sample preparation (SP3) protocol followed by TMT isobaric mass tagging as described previously [23].

Samples were desalted by BRAVO Assaymap (Agilent Technologies) using C18-cartridges, and pre-fractionated using high pH reverse-phase chromatography using an Ul6mate3000 RLSC (Dionex) as previously described [24]. All individual 24 fractions were directly loaded on an Ultimate 3000 coupled online to an Orbitrap Exploris. MS instrument was operated, and data was processed as described previously [24].

Log_2_ ion intensities were normalised and used as a measure for protein abundance. A linear model implemented in the *limma* package [25] was used for differential analysis. *p* values were adjusted for multiple testing as previously described [22] and proteins with an absolute log_2_ fold-change ≥ 1.5, adjusted *p*-value ≤ 0.05 classified as significantly differentially expressed.

### Colony forming assays

1000 cells/well were seeded of a 6-well plate in triplicate and incubated for 24 h, before the addition of either niraparib (0–40 µM), olaparib (0–50 µM), media alone or carrier control for 72 h. Media was removed, cells washed with PBS, fresh culture media added and incubated for 14 days, before colonies were fixed using 1 mL methanol for 30 min at room temperature and stained using 1 mL 0.5% crystal violet stain (0.5% w/v crystal violet, 25% v/v methanol) for 15 min. Plates were washed gently with dH_2_O and air-dried. Images were captured using a Canon imageRUNNER C5235i photocopier unit and colonies quantified by adding 1 mL 10% acetic acid to each well for 1 h at room temperature, with gentle rocking. Absorbance readings were measured at 595 nm using a Multiskan spectrophotometer (ThermoFisher), with data replicated in 3 independent experimental repeats.

### ABC transporter protein permeability transport assay

The ability of P-gp and BCRP to transport olaparib, niraparib and amprenavir (positive control) was compared using a quantitative bi-directional transwell assay in polarised MDCKII cell lines, and derivates engineered to overexpress P-gp or BCRP, provided by Professor Kevin Read, Drug Discovery Unit, School of Life Sciences, University of Dundee. Apical to basolateral (A to B) and basolateral to apical (B to A) passive permeability was assessed in the presence and absence of test drugs and the ABC transporter inhibitor elacridar. Cells were seeded (1 × 10^6^ cells per well) in 450 μL DMEM supplemented with 10% v/v FBS and 1% penicillin/streptomycin (1000 Units/mL and 1000 μg/mL, respectively) in a 24-well plate transwell chamber (Greiner, Gloucestershire, UK) and incubated for 72 h. Media was replaced and cells incubated for a further 1 h before substitution with PBS or PBS containing 2 μM elacridar and incubated again for 1 h. Transport assay dosing stock solutions were prepared containing 1 mM test compound (olaparib, niraparib or amprenavir), 100 mM Lucifer yellow, a non-substrate control to confirm membrane integrity (Sigma Aldrich, Dorset), UK, 1% DMSO ± 2 mM elacridar. 10 μL of each stock solution was then diluted 1:1000, giving final concentrations of 1 μM test compound, 100 μM Lucifer yellow, 1% DMSO ± 2 μM Elacridar (Solutions A and B). PBS was removed from both apical and basolateral chambers and 450 μL of solutions A or B added to apical and 1.3 mL to basolateral compartments (Fig. 5a) and plates returned to the incubator for 1 h, before transwell inserts were removed to prevent further transport. 75 μL solution from each chamber was transferred to a 96-well deep well mass spectrometry plate (Waters, Wilmslow, UK), and compared with 75 μL dosing solution (C_0_), and 200 μL internal standard (acetonitrile, 232 nM sulfadimethoxine). Ultra-high performance liquid chromatography tandem mass spectrometry (UPLC-MS/MS) on a Xevo TQ-S micro–Triple Quadrupole Mass Spectrometer (Waters, U.S.A) was used to analyse drug concentrations. An apparent permeability (Papp) score was calculated for each test compound for both A to B and B to A transport, in the presence and absence of elacridar. Comparison of efflux ratio (PappB-A/PappA-B) in the presence and absence of elacridar predicted whether a test compound was actively effluxed, with a fold change in efflux ratio ≥ 3 confirming substrate specificity.

### Copy number assays

*ABCB1* and *ABCG2* copy number was assessed in A2780, A2780pacR, A2780olapR and A2780nirapR cells using quantitative Taqman gene copy number assays (Taqman assay IDs Hs04962504_cn (*ABCB1*) and Hs003508853_cn (*ABCG2*)), where target gene copy number was compared with the copy number of the endogenous control gene RNAse P (Taqman Copy Number Reference Assay) by the comparative Ct method, and relative quantitation values obtained using CopyCaller Software (Life Technologies). As additional controls, copy number was assessed in peripheral blood samples (*n* = 2) obtained from healthy volunteers, and copy numbers of two confirmed.

### Statistical methods

Pairwise comparisons of experimental data (gene expression, protein expression or IC_50_ values were performed using students’ unpaired (cell lines) and paired *t* tests (DOCS study samples) using GraphPad PRISM 10, with *p* < 0.05 considered to represent statistical significance (* *p* < 0.05, ** *p* < 0.01, *** *p* < 0.001). Spearman’s rank correlation to compare *ABCB1* and *ABCG2* expression in DOCS study samples was performed using IBM SPSS Statistics v.29.0.2.0.

## Results

### Creation of a novel niraparib-resistant cell line model

**Fig. 1:**
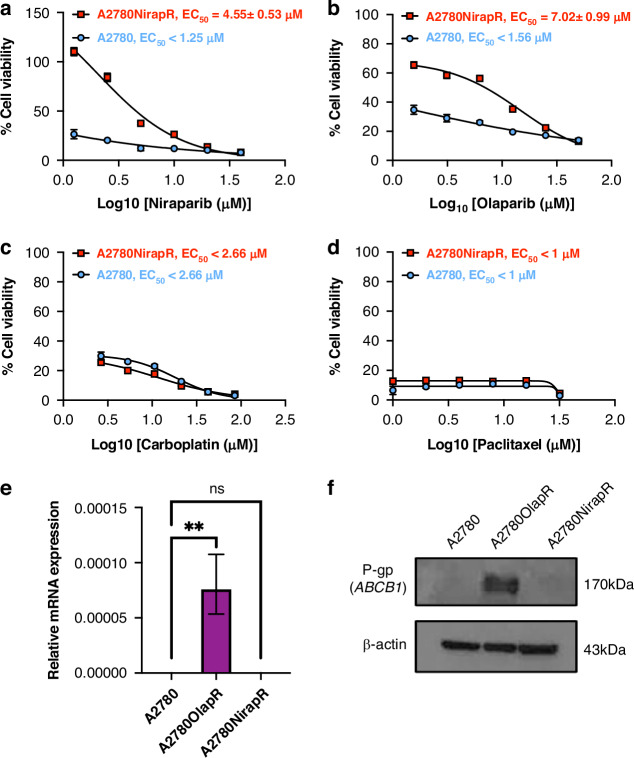
Creation of a novel niraparib-resistant cell line model, A2780nirapR. A2780 cells were continuously exposed to increasing concentrations of niraparib (0.1 µM–12.8 µM) until stable resistance was achieved (A2780nirapR). **a** Sensitivity to niraparib (1.25 µM–40 µM) was compared in A2780 and A2780nirapR cells by MTT chemosensitivity assay, and cross-resistance of A2780nirapR cells to (**b**) olaparib (1.56 µM–50 µM) (**c**) carboplatin (2.66 µM–85.12 µM) and (**d**) paclitaxel (1 µM–31.85 µM) was similarly assessed. *ABCB1* (P-gp) expression was compared in A2780, A2780olapR and A2780nirapR cells by (**e**) qRT-PCR analysis, relative to the expression of 18S ribosomal RNA and (**f**) Western blotting, relative to the expression of β-actin. Student’s unpaired *t* tests were used for pairwise comparisons in qRT-PCR analysis and MTT assays, where experiments were performed in triplicate and error bars represent compound standard deviation (qRT-PCR analysis) or standard deviation (MTT assays), as described in Methods. ** = *p* < 0.01, ns = not significant.

To compare resistance mechanisms following olaparib and niraparib treatment and to allow direct comparison with previously created A2780olapR cells [16], we created a novel niraparib resistant cell line (A2780nirapR) following continuous selection in increasing concentrations (0.1 µM–12.8 µM) of niraparib. A2780nirapR cells were resistant to 12.8 μM niraparib (>3.6-fold change in EC_50_, Fig. 1a), mimicking typical steady state peak plasma levels in ovarian cancer patients [20], and were cross-resistant to olaparib (>4.5-fold change in EC_50_, Fig. 1b), but not to carboplatin (Fig. 1c) or paclitaxel (Fig. 1d). Unlike A2780olapR cells, *ABCB1* mRNA (Fig. 1e) and P-gp protein (Fig. 1f) expression was not increased in A2780nirapR cells.

### A2780olapR and A2780nirapR cells have unique gene expression profiles

**Fig. 2:**
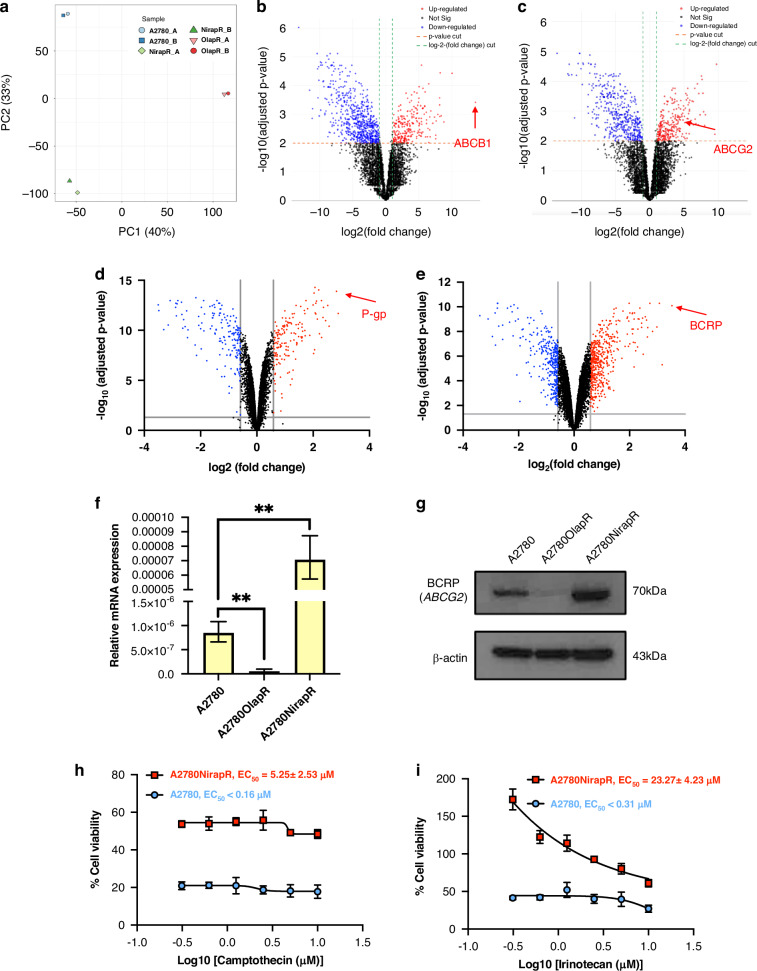
A2780olapR and A2780nirapR cells have unique gene expression profiles. RNASeq analysis (Ion Ampliseq^TM^ Transcriptome Human Gene expression analysis, ThermoFisher) was used to compare gene expression in duplicate replicates of A2780, A2780olapR and A2780nirapR cells. **a** Global changes in gene expression were illustrated in a Principal Component Analysis (PCA) plot, with differentially expressed genes (≥2-fold change in gene expression, adjusted *p* value ≤ 0.05) illustrated in Volcano plots following pairwise comparison of gene expression in A2780 and (**b**) A2780olapR (*ABCB1* highlighted) and (**c**) A2780nirapR (*ABCG2* highlighted) cells (upregulated genes are highlighted in red, down regulated genes in blue and unchanged genes in black). Similarly, LC-MS/MS proteomics analysis was used to identify differentially expressed proteins (≥1.5-fold change in protein expression, adjusted *p* value ≤ 0.05) in (**d**) A2780 and A2780olapR (P-gp highlighted) and (**e**) A2780 and A2780nirapR (BCRP highlighted) cells. Increased *ABCG2* expression in A2780nirapR cells was confirmed by (**f**) qRT-PCR analysis, relative to the expression of 18S ribosomal RNA and (**g**) Western blotting, relative to the expression of β-actin. Cross-resistance of A2780nirapR cells to (**h**) camptothecin and (**i**) irinotecan was confirmed by MTT chemosensitivity assays. Student’s unpaired *t* tests were used for pairwise comparisons in qRT-PCR analysis and MTT assays, where experiments were performed in triplicate and error bars represent compound standard deviation (qRT-PCR analysis) or standard deviation (MTT assays), as described in Methods. ** = *p* < 0.01.

RNASeq analysis was used to perform an unbiased comparison of gene expression in duplicate replicates of A2780, A2780olapR and A2780nirapR cells. Principal Component Analysis (PCA) of the RNASeq dataset confirmed reproducibility of experimental replicates, and highlighted distinct gene expression clusters for each cell line (Fig. 2a), suggesting that A2780olapR and A2780nirapR cells may have become resistant by distinct mechanisms. 1715 genes were differentially expressed (≥2-fold change, adjusted *p* value ≤ 0.05) in A2780olapR cells (536 up-regulated and 1179 down-regulated) and 1572 genes (682 up-regulated and 890 down-regulated) in A2780nirapR cells. Functional enrichment analysis of differentially expressed genes identified multiple differences in KEGG pathways and GO terms in A2780olapR and A2780nirapR cells (Supplementary Table 1), suggesting that divergent resistance mechanisms result from olaparib and niraparib selection. Focussing on ABC transporter expression (additional prioritised candidate genes will be discussed in a second manuscript) and consistent with our previous data, *ABCB1* expression was significantly increased in A2780olapR cells (Fig. 2b, fold change 12070.24, adjusted *p* value = 0.0004; Supplementary Table 2), with a more modest increase in *ABCB4* expression (fold change 12.50, adjusted *p* value = 0.03), while the expression of *ABCG1* (fold change −14.55, adjusted *p* value = 0.001), *ABCG2* (fold change −10.89, adjusted *p* value = 0.004) and *ABCA5* (fold change −2.51, adjusted *p* value = 0.04) was decreased. In contrast, *ABCB1* was not differentially expressed in A2780nirapR cells, where expression of the alternative ABC transporter *ABCG2* was significantly increased (Fig. 2c, fold change 80.75, adjusted *p* value = 0.0005), with a more modest increase in *ABCA5* expression (fold change 4.43, adjusted *p* value = 0.009), and decreased expression of *TAP1* (fold change −7.03, adjusted *p* value = 0.0002) and *ABCG1* (fold change −13.21, adjusted *p* value = 0.002). Differential drug transporter expression profiles in A2780olapR and A2780nirapR cells was further confirmed by unbiased LC-MS/MS proteomics analysis, where P-gp was the only drug transporter significantly induced in A2780olapR cells (fold change 2.83, adjusted *p* value = 1.29 × 10^−14^; Fig. 2d) and BCRP in A2780nirapR cells (fold change 3.53, adjusted *p* value = 7.97 × 10^−11^; Fig. 2e). Induction of *ABCG2* expression in A2780nirapR cells was further confirmed by qRT-PCR (Fig. 2f) and Western blot (Fig. 2g) analysis—in contrast, *ABCG2* expression was consistently repressed in A2780olapR cells in RNASeq, qRT-PCR and Western blot analysis. As predicted, A2780nirapR cells were cross-resistant to the well characterised BCRP substrates irinotecan (Fig. 2h) and camptothecin (Fig. 2i).

### Loss of *ABCG2* expression re-sensitises A2780nirapR cells to niraparib and olaparib

**Fig. 3:**
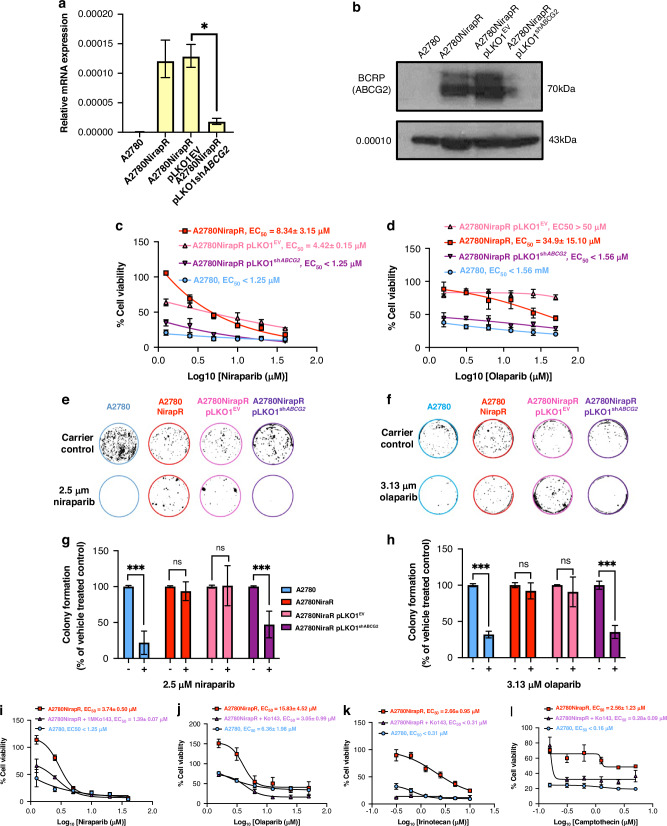
Loss of ABCG2 expression re-sensitises A2780nirapR cells to niraparib and olaparib. A control (pLKO1^EV^) plasmid and shRNA plasmid targeting *ABCG2* (pLKO1^ABCG2^) were introduced into A2780nirapR cells, with the influence on *ABCG2* and BRCP expression assessed by (**a**) qRT-PCR analysis, relative to the expression of 18S ribosomal RNA and (**b**) Western blotting, relative to the expression of β-actin. The influence of introducing pLKO1^EV^ and sh*ABCG2* plasmids on chemosensitivity to (**c**) niraparib and (**d**) olaparib was assessed by MTT assays, and colony formation assays following challenge with (**e**, **g**) niraparib and (**f**, **h**) olaparib. The influence of combination treatment with the BCRP inhibitor Ko143 on chemosensitivity to (**i**) niraparib, (**j**) olaparib, (**k**) irinotecan and (**l**) camptothecin in A2780nirapR cells was further assessed by MTT assays. Student’s unpaired *t* tests were used for pairwise comparisons in qRT-PCR analysis and MTT assays, where experiments were performed in triplicate and error bars represent compound standard deviation (qRT-PCR analysis) or standard deviation (MTT assays), as described in Methods. * = *p* < 0.05, *** = *p* < 0.001, ns = not significant.

To investigate whether loss of *ABCG2* expression altered chemosensitivity in A2780nirapR cells, we used an *ABCG2*-targetted Mission shRNA construct to stably knock down *ABCG2* expression. Transfection of an empty vector (EV) control (A2780NirapR pLKO1^EV^) did not significantly change *ABCG2* expression, while introduction of shRNA *ABCG2* led to > 85% reduction in *ABCG2* mRNA expression (A2780NirapR pLKO1^shABCG2^, Fig. 3A), and complete loss of BRCP protein expression (Fig. 3B).

MTT assays were used to compare chemosensitivity to niraparib (Fig. 3c) and olaparib (Fig. 3d) in A2780, A2780nirapR, A2780NirapR pLKO1^EV^ and A2780NirapR pLKO1^shABCG2^ cells, where loss of *ABCG2* led to re-sensitisation of A2780nirapR pLKO1^shABCG2^ cells. Similar results were obtained using clonogenic assays to assess the ability of niraparib (Fig. 3e, g) and olaparib (Fig. 3f, h) to influence colony formation. Colony formation was significantly increased in A2780nirapR, and reduced in A2780NirapR pLKO1^shABCG2^ cells in the presence of either drug in comparison to A2780 control cells, suggesting that *ABCG2* knockdown restores a drug-sensitive phenotype.

To confirm that the drug-resistant phenotype in A2780NirapR cells can also be influenced by a combination drug treatment approach, we combined niraparib or olaparib with Ko143, a small molecule BCRP-selective inhibitor. Combination treatment with 1 µM Ko143 re-sensitised A2780NirapR cells to both niraparib (Fig. 3i) and olaparib (Fig. 3j) and also, as expected, to the well-characterised BCRP substrates irinotecan (Fig. 3k) and camptothecin (Fig. 3l).

### Expression of *ABCG2* in A2780 cells creates a drug-resistant phenotype

**Fig. 4:**
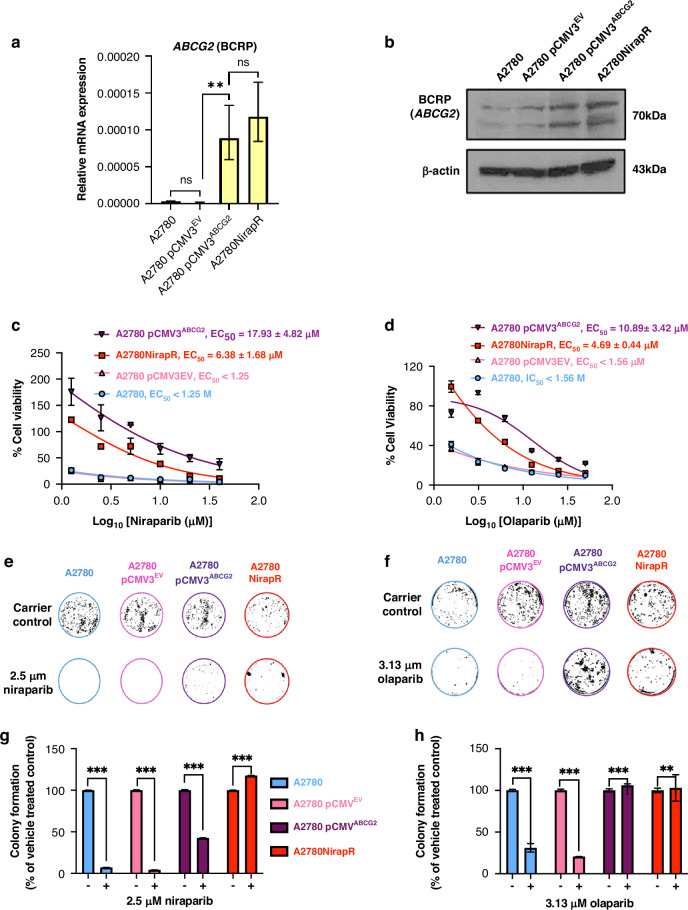
Expression of ABCG2 in A2780 cells creates a drug-resistant phenotype. A pCMV3^EV^ control plasmid and plasmid expressing *ABCG2* was introduced into A2780nirapR cells, with the influence on *ABCG2* and BRCP expression assessed by (**a**) qRT-PCR analysis, relative to the expression of 18S ribosomal RNA and (**b**) Western blotting, relative to the expression of β-actin. The influence of pCMV3^EV^ and pCMV3^ABCG2^ plasmid incorporation on chemosensitivity to (**c**) niraparib and (**d**) olaparib was assessed by MTT assays and colony formation assays following challenge with (**e**, **g**) niraparib and (**f**, **h**) olaparib. Student’s unpaired *t* tests were used for pairwise comparisons in qRT-PCR analysis and MTT assays, where experiments were performed in triplicate and error bars represent compound standard deviation (qRT-PCR analysis) or standard deviation (MTT assays), as described in Methods. ** = *p* < 0.01, *** = *p* < 0.001, ns = not significant.

To assess the phenotypic consequences of gain of *ABCG2* expression, *ABCG2* was heterologously expressed in drug-sensitive A2780 cells (A2780 pCMV3^ABCG2^). *ABCG2* and BCRP expression, assessed by qRT-PCR (Fig. 4a) and Western blot analysis (Fig. 4b), respectively, was similar to expression in A2780nirapR cells, allowing a direct comparison of chemosensitivity in transfected and drug-resistant cells. A2780 cells became resistant to both niraparib (Fig. 4c) and olaparib (Fig. 4d) when *ABCG2* was expressed. Similar results were obtained using clonogenic assays to assess the ability of niraparib (Fig. 4e, g) and olaparib (Fig. 4f, h) to influence colony formation. Colony formation was significantly higher and  was not influenced by drug treatment in A2780 pCMV3^ABCG2^ cells and A2780nirapR cells, in contrast to reduced colony formation and drug sensitivity in A2780 and A2780 pCMV3 control cells.

### Both P-gp and BCRP actively efflux olaparib and niraparib

**Fig. 5:**
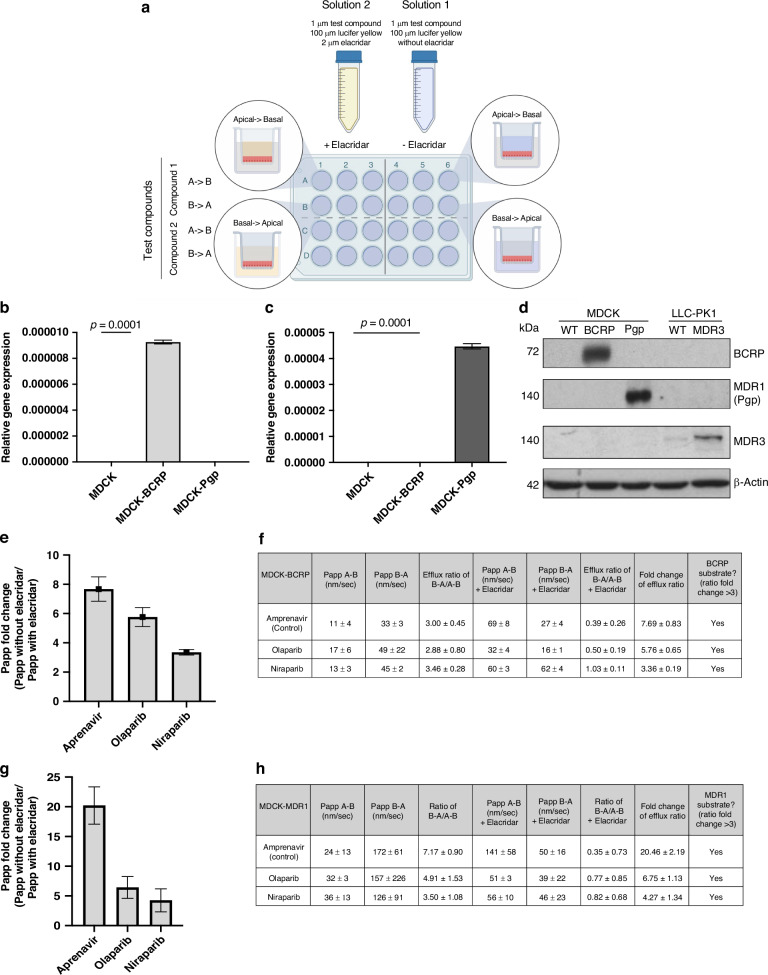
Both P-gp and BCRP actively efflux olaparib and niraparib. **a** Transwell ABC transporter permeability assays in polarised MDCK-II and derivative cell lines stably over-expressing P-gp and BCRP were used to compare the ability of triplicate replicates of each cell line to efflux niraparib, olaparib and the control substrate amprenavir in the presence and absence of the P-gp/BRCP inhibitor elacridar. Parental MDCK-II cells did not express either transporter, with expression of (**b**) *ABCG2* in MDCK-BRCP cells (**c**) *ABCB1* in MDCK-Pgp cells confirmed by qRT-PCR and (**d**) Western blot analysis. Ultra-high performance liquid chromatography tandem mass spectrometry (UPLC-MS/MS) was used to analyse drug concentrations following drug incubation in apical and basal chambers, and an apparent permeability (Papp) score calculated for (**e**, **f**) niraparib and (**g**, **h**) olaparib, in the presence and absence of elacridar, where a final fold change in efflux ratio ≥ 3 confirmed active transport. Errors represent standard deviation or compound standard deviation where appropriate, as described in Methods.

To investigate substrate selectivity for P-gp and BCRP, we used transwell ABC transporter passive permeability assays in polarised MDCK-II and derivative cell lines stably over-expressing P-gp and BCRP to compare drug efflux. Parental MDCK-II cells do not express either drug transporter, while cells engineered to over-express P-gp or BCRP show reduced intracellular drug concentrations when exposed to a drug which is actively effluxed. As both P-gp and BCRP are efflux transporters, basal->apical transport is predicted to be greater than apical->basal transport, and drug efflux is reduced in the presence of the common P-gp and BCRP inhibitor elacridar (Fig. 5A). Cell line specific expression of BCRP and P-gp was confirmed in MDCK-BCRP (Fig. 5b, d) and MDCK-P-gp (Fig. 5c, d) cells, by qRT-PCR and Western blot analysis. Ultra-high performance liquid chromatography tandem mass spectrometry (UPLC-MS/MS) was used to analyse drug concentrations and an apparent permeability (Papp) score calculated for niraparib and olaparib, in the presence and absence of elacridar, where a final efflux ratio ≥3 confirmed active transport. The protease inhibitor amprenavir is actively transported by both P-gp and BCRP, and was included as an additional assay validation positive control. As expected, amprenavir was transported by both BRCP (Fig. 5e, f) and P-gp (Fig. 5g, h). Olaparib (efflux ratio 5.75 ± 0.65) and niraparib (efflux ratio 3.35 ± 0.19) were both effluxed by BCRP, with P-gp transporting olaparib (efflux ratio 6.42 ± 1.83) more efficiently than niraparib (efflux ratio 4.26 ± 1.93), which did not meet the efflux ratio ≥3 threshold within experimental error.

### Drug transporter expression is variable in primary ovarian cancer cells, and is consistently induced in drug-resistant patients

**Fig. 6:**
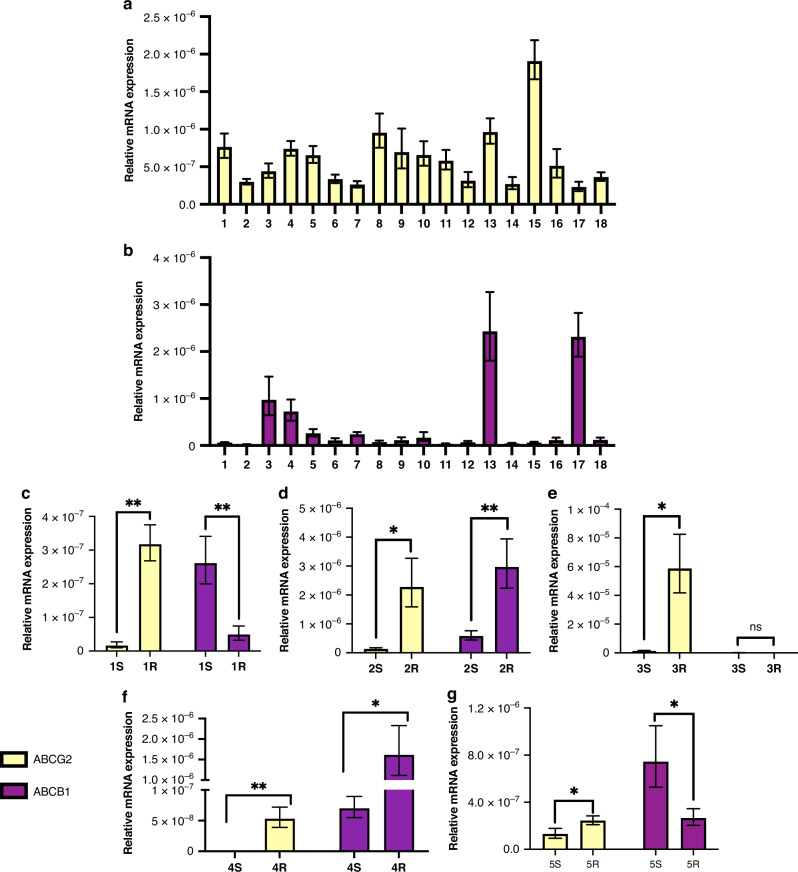
ABCG2 expression is preferentially induced in niraparib-resistant DOCS study patients. qRT-PCR analysis was used to compare (**a**) *ABCG2* (yellow) and (**b**) *ABCB1* (purple) expression in ascites-derived primary cell lines created from drug-resistant ovarian cancer patients (*n* = 18) recruited to the DOCS study, and (**c**–**g**) in a subset of 5 matched ascites-derived primary cell lines created from initially drug-sensitive and subsequently drug-resistant DOCS study patients. Student’s paired *t* tests were used for pairwise comparisons, where experiments were performed in triplicate and error bars represent compound standard deviation, as described in Methods. * = *p* < 0.05, ** = *p* < 0.01.

To confirm that *ABCG2* and *ABCB1* were expressed in clinical samples, qRT-PCR analysis was used to quantify *ABCG2* (Fig. 6a) and *ABCB1* expression (Fig. 6b) in ascites-derived primary cell lines created from 18 drug-resistant DOCS study ovarian cancer patients. *ABCG2* and *ABCB1* expression was variable and did not correlate (Spearman’s rho = 0.03, *p* = 0.906), consistent with our RNASeq analysis predictions of alternative mechanisms of gene regulation. Focussing on a subset of 5 DOCS study patients where matched drug-sensitive chemotherapy naïve and drug-resistant primary cell lines were available from niraparib-treated patients, *ABCG2* expression was consistently increased in all resistant patients, assessed by qRT-PCR analysis (Fig. 6c–g). In contrast, *ABCB1* expression was increased in only 2 patients (Fig. 6d, f), both of whom had been pre-treated with paclitaxel.

## Discussion

Olaparib and niraparib are increasingly prescribed as maintenance therapy in ovarian cancer patients, although the onset of adaptive resistance frequently impacts response [10]. Diverse resistance mechanisms, including restoration of homologous recombination repair (HRR, [17]), creation of novel fusion genes [26, 27] or increased expression of P-gp [16, 28] have been described for olaparib, but it was not known whether patients become resistant to niraparib by similar mechanisms. To address this question, we developed novel olaparib and niraparib-resistant cell line models to characterise resistance mechanisms using both candidate gene and unbiased RNASeq and proteomics approaches.

Importantly, we confirmed that PARPi-resistant cells were not cross-resistant to either carboplatin or paclitaxel, suggesting that maintenance PARPi treatment may not limit further use of these cytotoxic drugs. Principal component analysis of differentially expressed genes in A2780olapR and A2780nirapR cells confirmed that resistant cell lines were phenotypically distinct, with functional enrichment analysis identifying multiple differences in KEGG pathways and GO terms. Although e.g. the KEGG pathway for cell adhesion molecules was common to both cell lines, divergent resistance mechanisms were induced following olaparib and niraparib selection, with olaparib-resistant cells showing enrichment of MAPK and VEGF signalling and niraparib-resistant cells enrichment of p53 signalling and the central carbon metabolism of cancer.

Since the first association of P-gp with drug resistance [14], altered expression of ABC efflux transporters including *ABCG2* (BCRP) and *ABCC1* (MRP1) has been associated with the development of adaptive drug resistance in multiple cancers [29]. We have shown that paclitaxel-induced P-gp overexpression promoted cross-resistance to olaparib and that resistance was PARPi-specific, with paclitaxel inducing cross-resistance to olaparib and rucaparib, but not to veliparib or AZD2461, a modified congener of olaparib, designed to limit P-gp interactions [16, 30]. Consistent with our findings, Lawlor et al. had previously reported that olaparib but not veliparib was a P-gp substrate in IGROVCDDP and KB-8-5-11 cells [31], while prescribing information from the Food and Drug Administration (FDA) confirms that olaparib is both a BCRP substrate and inhibitor [32].

We have recently confirmed that A2780pacR cells are cross-resistant to niraparib, where resistance was reversible by co-administration of verapamil, elacridar, and tariquidar, suggesting that niraparib may also be a P-gp substrate (Amber McWhirter, personal communication). Unexpectedly, however, P-gp expression was not induced in A2780nirapR cells, which uniquely expressed the alternative efflux transporter BCRP. Extending our candidate gene analysis to whole genome RNASeq and proteomics analysis, we further confirmed that *ABCB1*/P-gp was the most abundantly over-expressed gene in A2780olapR cells but was not differentially expressed in A2780nirapR cells, where *ABCG2*/BCRP was the most abundantly expressed drug efflux transporter. Intriguingly, *ABCB1*/P-gp expression was decreased in A2780nirapR cells, suggesting that drug resistance may result from both positive and negative regulation of drug efflux transport activity. Recent data from Teng et al. describes a similar mechanism of BCRP-mediated resistance in A2780 cells resistant to the alternative PARPi talazoparib. Talazoparib-resistant cells (A2780/T4) had increased *ABCG2* expression, and were cross-resistant to the BCRP substrates mitoxantrone and topotecan. *ABCG2* knockdown re-sensitised cells to talazoparib, while *ABCB1* loss did not modify chemosensitivity [33].

shRNA-mediated *ABCG2* knockdown re-sensitised A2780nirapR cells to both niraparib and olaparib, suggesting that both drugs may be BCRP substrates. Consistent with this hypothesis, heterologous expression of *ABCG2* in drug-sensitive A2780 cells induced a drug-resistant phenotype, with *ABCG2*-overexpressing cells resistant to both olaparib and niraparib. Using a quantitative bi-directional transwell assay in polarised MDCKII cell lines, we confirmed that both BCRP and P-gp can transport olaparib and niraparib, although P-gp exhibits greater selectivity for olaparib. Our data is consistent with previous studies of PARPi bioavailability in mouse models, which suggested that niraparib is a substrate for both murine P-gp and Bcrp [34], P-gp had greater affinity for olaparib than Bcrp [35], and niraparib had greater affinity for Bcrp than olaparib [1].

Importantly, our data suggests that drug-induced transcriptional regulation of drug transporter expression, where olaparib induces *ABCB1* expression and niraparib *ABCG2*, is a more important modifier of the drug resistance phenotype than substrate specificity. Consistent with this hypothesis, and in contrast to our previous report confirming increased *ABCB1* copy number in paclitaxel-resistant A2780pacR cells [16], we recently confirmed that the development of adaptive resistance to olaparib and niraparib does not promote amplification of *ABCB1* or *ABCG2* (Supplementary Fig. 1).

Co-treatment of niraparib or olaparib with the BCRP-selective inhibitor Ko143 re-sensitised A2780nirapR cells, suggesting that future prescription of active drug with a resistance pathway inhibitor may postpone or prevent the development of drug resistance in ovarian cancer patients. We highlight the need to prioritise future development and clinical evaluation of BCRP-specific inhibitors with improved oral bioavailability, acceptable toxicity profiles and blood-brain barrier permeability. Makrecka-Kuka et al. describe an interesting approach where olaparib was conjugated with selenophenoquinolinone derivatives which maintain PARP1 inhibition while inhibiting *ABCB1* transporter activity [36], and it is interesting to note that Li et al. describe a novel flavonoid BCRP inhibitor, marein, which re-sensitised resistant COV362 cells to various drugs including olaparib [2]. Niraparib was not tested, however, and these investigators did not compare inhibitor specificity for BCRP and P-gp. Although novel inhibitors with improved substrate specificity are urgently required, our DOCS study data highlights the potential clinical utility of pan-transporter inhibitors, particularly in patients treated sequentially with paclitaxel and PARPis.

Expression of *ABCG2* was consistently induced in matched primary DOCS study ascites-derived cell lines, suggesting that we have identified a clinically relevant resistance mechanism. Whilst *ABCG2* induction has been previously described in drug-resistant cell line models [29], to the best of our knowledge, this is the first report to identify increased *ABCG2* expression as a biomarker of niraparib resistance in ovarian cancer patients.

Increased expression of drug efflux transporters is consistent with the hypothesis that adaptive drug resistance results from the expansion of an inherently resistant stem cell population [37]. Yan et al. described biological characteristics of drug-resistant ovarian cancer cells overexpressing CD133, CD117, Nanog, Oct4, and *ABCG2* [38], while Jung et al. and Yin et al. described BCRP up-regulation in doxorubicin-resistant A2780 cells as a consequence of increased HSP90 regulation of PIK3CA/AKT/c-Met signalling [39, 40]. An alternative mechanism of BCRP activation in ovarian cancer stem cells was proposed by He et al., who suggested that HIF-2α transcriptionally activates BCRP, leading to increased “stemness” and poor outcomes in ovarian cancer patients [41].

In contrast to *ABCG2*, which was consistently induced in niraparib-resistant patients, increased *ABCB1* expression was only observed in patients pre-treated with paclitaxel. Our data therefore suggests that both drug choice and scheduling of primary chemotherapy is likely to influence resistance mechanisms. Continued routine inclusion of paclitaxel in first-line combination chemotherapy for ovarian cancer may promote resistance to both olaparib and niraparib by induction of P-gp, suggesting that single-agent carboplatin may be a more appropriate choice, particularly in BRCA1/2 mutant or HRD patients who respond well to carboplatin and receive the greatest benefit from subsequent maintenance PARPi therapy. Unfortunately, our data confirms that PARPi maintenance treatment is likely to be compromised by adaptive resistance, but identifies P-gp and BCRP as novel drug-specific resistance biomarkers. Prospective studies on the clinical utility of ABC transporter biomarkers are urgently required, not only in ovarian cancer, but in other cancers including breast and prostate cancer, where PARPis are also licensed and increasingly prescribed [42, 43]. It is of particular interest to note that, similar to our own findings, docetaxel-resistant castration-resistant prostate cancer models were cross-resistant to olaparib as a consequence of increased P-gp expression, when resistance was reversible following combination treatment with elacridar or enzalutamide [3]. Finally, we highlight the urgent clinical need to identify and evaluate additional novel PARPi drug candidates which are not substrates for either P-gp or BCRP, to limit the development of adaptive drug resistance.

## Supplementary information

Supplementary Figure legends

KEGG pathway and GO term comparison of differentially expressed genes in A2780olapR and A2780nirapR cells

Summary of differential ABC transporter expression in A2780olapR and A2780nirapR cells, identified by RNASeq analysis

Supplementary Figure 1

## Data Availability

Analysed RNASeq and proteomics datasets are being prepared for related publications, and are available from the corresponding author upon reasonable request.
