# Supplementary material for: Identification of novel drug-specific PARP inhibitor resistance mechanisms in ovarian cancer–implications for clinical practice
Source: Br J Cancer. 2026 Apr 17;135(2):290–302. doi: 10.1038/s41416-026-03423-z (PMC13310856; doi:10.1038/s41416-026-03423-z)
Supplement: Supplementary file 1 — Supplementary Figure legends [file 41416_2026_3423_MOESM1_ESM.docx]

Supplementary Table 1: KEGG pathway and GO term comparison of differentially expressed genes in A2780olapR and A2780nirapR cells

KEGG pathway and GO term analysis was performed by FIOS Genomics, following RNASeq analysis of A2780, A2780olapR and A2780nirapR cells and identification of differentially expressed genes (≥2-fold change in gene expression, adjusted p-value≤0.05). Using the same thresholds in pathway and process enrichment analysis, 4 KEGG pathways were enriched in A2780olapR cells and 27 pathways in A2780nirapR cells. All 4 A2780olapR pathways were common to A2780nirapR cells (highlighted in *italics*), which showed additional phenotypic diversity. 80 GO terms were enriched in A2780olapR cells and 93 in A2780nirapR cells, with only 17 terms in common, consistent with development of a core PARPi-resistance signature, but additional diversity in response to olaparib and niraparib challenge.

Supplementary Table 2: Summary of differential ABC transporter expression in A2780olapR and A2780nirapR cells, identified by RNASeq analysis

Differentially expressed ABC transporters were identified by FIOS Genomics in A2780olapR and A2780nirapR cells following pairwise comparisons of RNASeq data. Candidate genes meeting the differential expression threshold of ≥2-fold change in gene expression, adjusted p-value≤0.05, are highlighted in italics, with a significant induction in *ABCB1* and decrease in *ABCG2* expression highlighted in A2780olapR cells. In contrast, *ABCB1* was not differentially expressed in A2780nirapR cells, where *ABCG2* expression was significantly increased.

Supplementary Figure 1: *ABCG2* and *ABCG2* are not amplified in A2780olapR or A2780nirapR cells

A quantitative Taqman gene copy number assay was used to compare *ABCG2* (yellow) and *ABCB1* (purple) copy number in A2780, A2780olapR and A2780nirapR cells, where *ABCG2* copy number was compared with the copy number of the endogenous control gene *RNAse P* (Taqman Copy Number Reference Assay) by the comparative Ct method, and relative quantitation values obtained using CopyCaller Software. *ABCG2* and *RNAse P* copy numbers were additionally assessed in peripheral blood samples (n=2) obtained from healthy volunteers, where diploid copy number had been previously confirmed (14). Error bars represent compound standard deviation, relating quadruplicate assessments of *ABCG2* and *ABCG2* copy number to *RNAse P* copy number.
