# Supplementary figures and images for: Identification of novel drug-specific PARP inhibitor resistance mechanisms in ovarian cancer–implications for clinical practice
Source: Br J Cancer. 2026 Apr 17;135(2):290–302. doi: 10.1038/s41416-026-03423-z (PMC13310856; doi:10.1038/s41416-026-03423-z)

Supplementary Figure 1

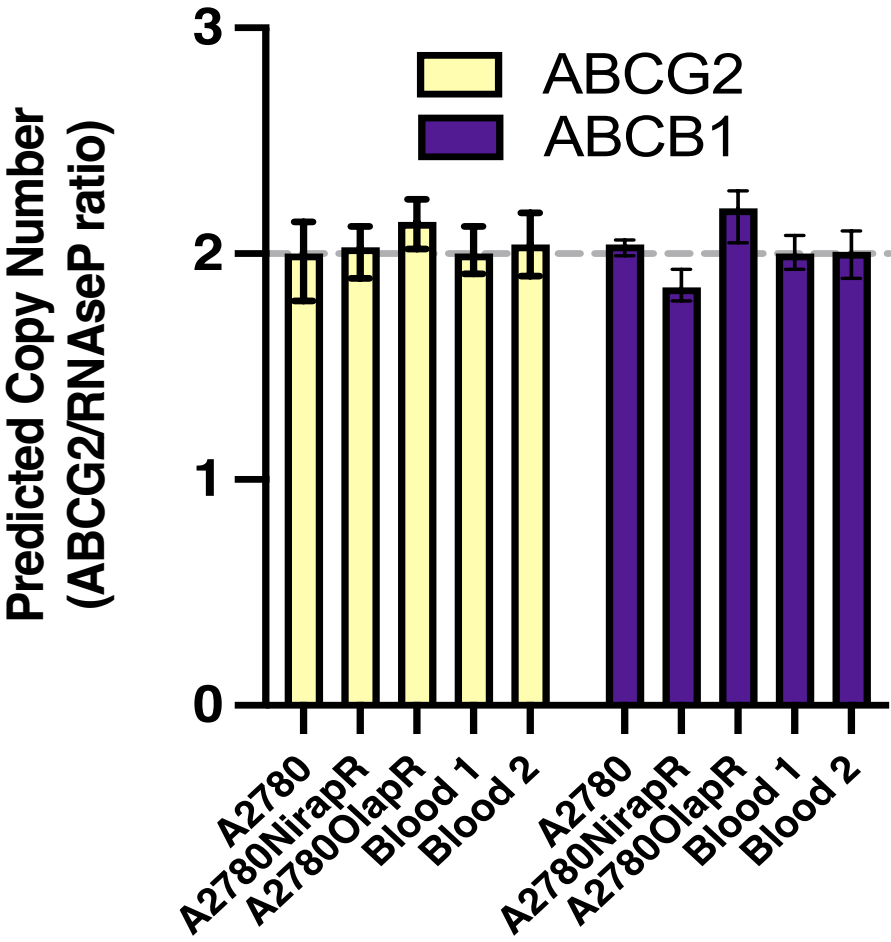

Supplement: Supplementary file 4 — Supplementary Figure 1 [file 41416_2026_3423_MOESM4_ESM.pdf]
